# Supplementary material for: Expansion of Kuravirus-like Phage Sequences within the Past Decade, including Escherichia Phage YF01 from Japan, Prompt the Creation of Three New Genera
Source: Viruses. 2023 Feb 11;15(2):506. doi: 10.3390/v15020506 (PMC9965538; doi:10.3390/v15020506)
Supplement: Supplementary file 1 [file viruses-15-00506-s001.zip › Supplementary Figures.pdf]

Supplementary Figure S1

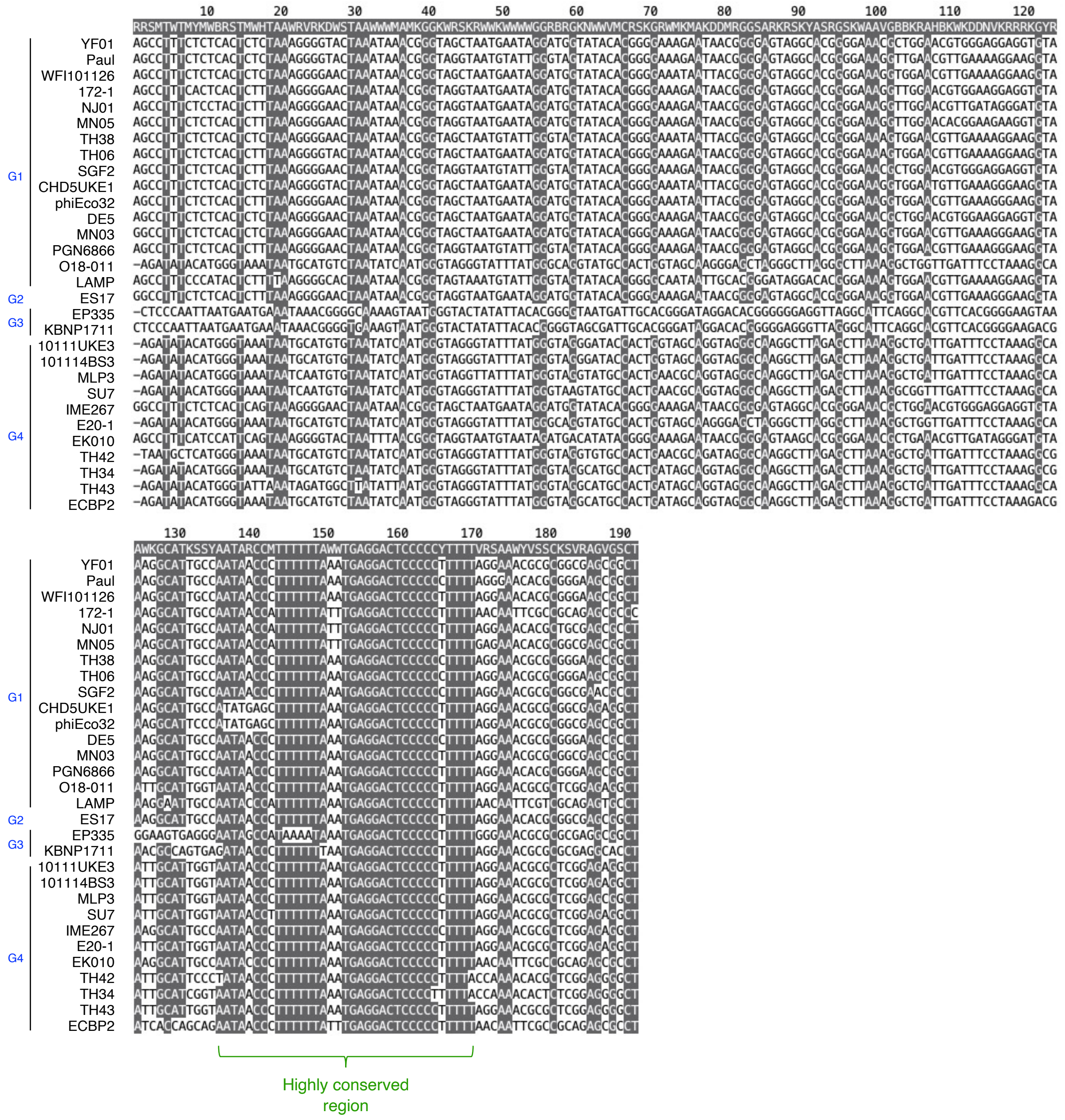

Supplementary Figure S1. The direct terminal repeat (DTR) sequence in *Kuravirus*-like cluster phages. The DTR sequences of YF01 phage was identified by inspection of raw long-reads (193 bp) and used to identity DTRs in other Tyroviruses by sequence comparison. Alignment of DTRs shows a regions of high sequence identity between nucleotide positions ~130-170. Size markers represent nucleotides.

Supplementary Figure S2

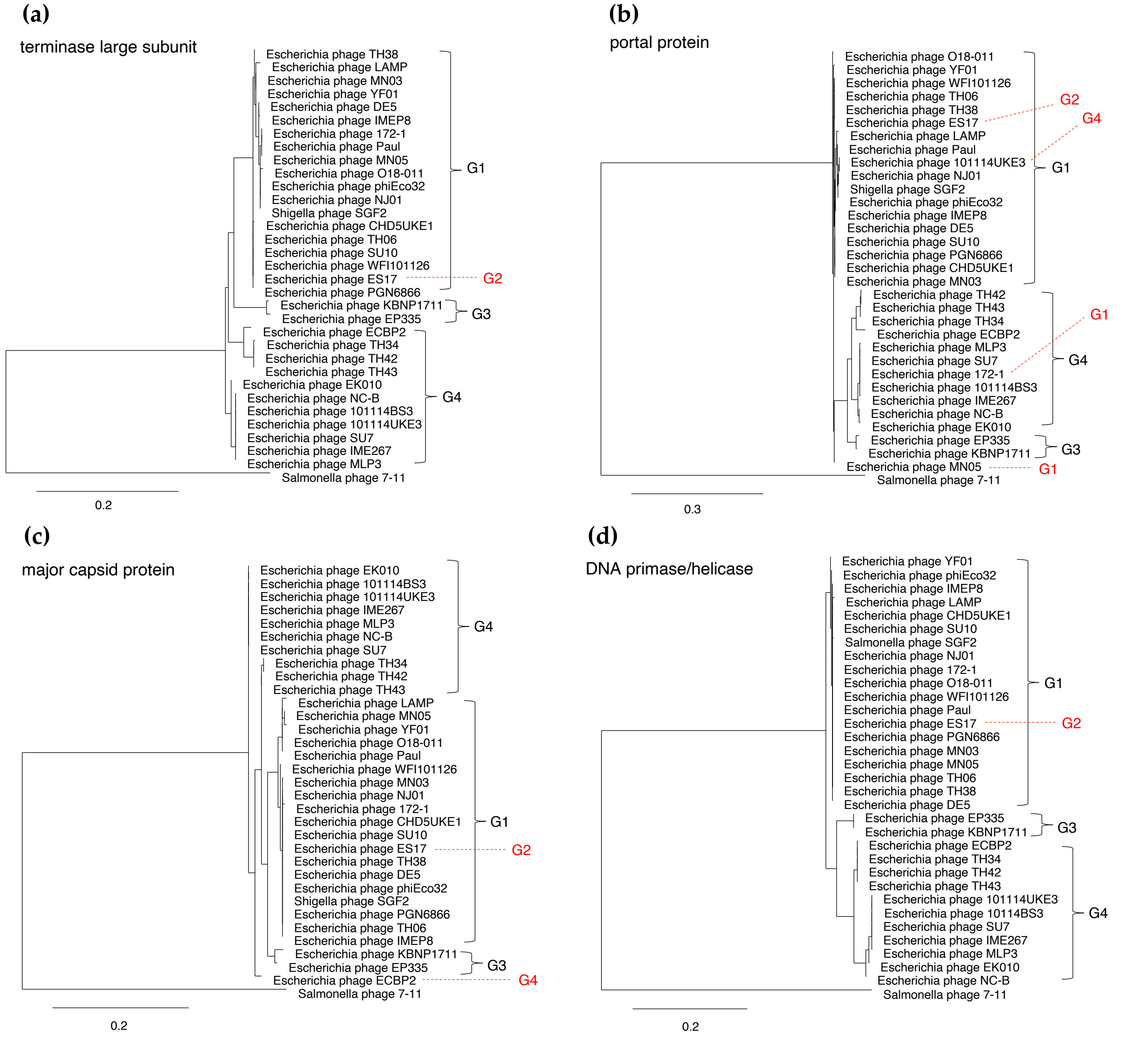

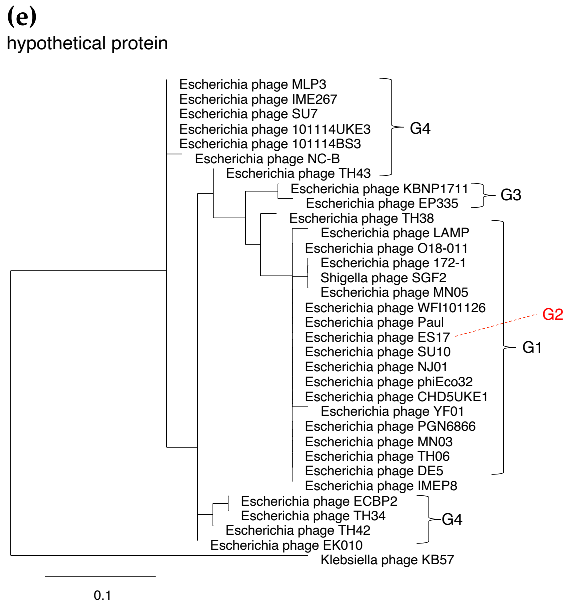

Supplementary Figure S2. Phylogenetic reconstruction based on five high-identity *Kuravirus*-like phage cluster core proteins. Phylogenetic reconstruction based on **a)** terminase large subunit, **b)** portal protein, **c)** major capsid protein, **d)** DNA primase/ helicase and **e)** a hypothetical protein, using the maximum likelihood method. Homologous proteins from the closely related *Salmonella* phage 7-11 or *Klebsiella* phage KB57, were used as out-groups. Scale bars represent the number of amino acid substitutions per site.
